# Non-invasive versus arterial pressure monitoring in the pre-hospital critical care environment: a paired comparison of concurrently recorded measurements

**DOI:** 10.1186/s13049-024-01240-y

**Published:** 2024-08-27

**Authors:** Yani Perera, James Raitt, Kurtis Poole, David Metcalfe, Asher Lewinsohn

**Affiliations:** 1 Thames Valley Air Ambulance, Stokenchurch House, Oxford Road, Stokenchurch, HP14 3SX Buckinghamshire UK; 2 Frimley Health NHS Foundation Trust, Camberley, Surrey UK; 3 Oxford Trauma & Emergency Care (OxTEC), University of Oxford, Oxford, UK; 4 Emergency Medicine Research Oxford (EMROx), John Radcliffe Hospital, Oxford, UK; 5 Bedfordshire Hospitals NHS Foundation Trust, Luton, Bedfordshire UK

**Keywords:** Blood pressure monitoring, Arterial line, Pre-hospital critical care

## Abstract

**Background:**

Blood pressure monitoring is important in the pre-hospital management of critically ill patients. Non-invasive blood pressure (NIBP) measurements are commonly used but the accuracy of standard oscillometric cuff devices may be affected by extremes of physiology and adverse conditions (e.g. vibration) during transport. This study aimed to quantify the accuracy of NIBP measurements amongst patients requiring pre-hospital critical care.

**Methods:**

A retrospective cohort study was undertaken using data from patients treated by a pre-hospital critical team between 1st May 2020 and 30th April 2023 that had NIBP measured concurrently with invasive blood pressure (IBP) arterial manometry. An acceptable difference was determined a priori to be < 20mmHg for systolic blood pressure (SBP) and diastolic blood pressure (DBP), and < 10mmHg for mean arterial pressure (MAP). The primary outcome was “pairwise agreement”, i.e. the proportion of paired observations that fell within this range of acceptability. Bland-Altman plots were constructed together with 95% limits of agreement to visualise differences between pairs of data. Associations with patient age, reason for critical care, transport status, haemodynamic shock, severe hypertension, and arterial catheter position were explored in univariate analyses and by fitting multivariable logistic regression models.

**Results:**

There were 2,359 paired measurements from 221 individual patients with a median age of 57. The most frequent reason for transport was cardiac arrest (79, 35.7%). Bland-Altman analyses suggested unacceptably wide limits of agreement with NIBP overestimating both SBP and MAP during hypotension and underestimating these values during hypertension. Haemodynamic shock (SBP < 90mmHg) was independently associated with reduced pairwise agreement for SBP (adjusted odds ratio [aOR] 0.52, 95% CI 0.35 to 0.77), DBP (aOR 0.65, 95% CI 0.42 to 0.99) and MAP (aOR 0.53, 95% CI 0.36 to 0.78) and severe hypertension (SBP > 160mmHg) with reduced pairwise agreement for SBP (aOR 0.17, 95% CI 0.11 to 0.27). There was no association between patient transport and agreement between the methods for SBP, DBP, or MAP.

**Conclusions:**

Non-invasive blood pressure measurements are often inaccurate in the pre-hospital critical care setting, particularly in patients with haemodynamic instability. Clinicians should be cautious when interpreting NIBP measurements and consider direct arterial pressure monitoring when circumstances allow.

**Supplementary Information:**

The online version contains supplementary material available at 10.1186/s13049-024-01240-y.

## Introduction

Blood pressure monitoring is important in the management of critically ill patients. It is most commonly measured using non-invasive blood pressure (NIBP) techniques such as disposable oscillometric cuff devices [1]. These require little training to use, are quick to apply, and do not expose the patient to risks of bleeding or infection [2]. However, these devices typically measure blood pressure intermittently, which may lead to delayed recognition and treatment of rapidly deteriorating patients. There is also evidence from a range of inpatient settings that oscillometric NIBP techniques underestimate extremes of blood pressure [3–6] and from the aeromedical literature that their accuracy is affected by vibration during transport [7, 8].

The standard of care for critically ill patients in hospital is direct intra-arterial blood pressure monitoring [9, 10]. These devices are more expensive, difficult to set up, and expose the patient to risks of bleeding and infection [1]. However, they provide continuous blood pressure monitoring and so can guide resuscitation on a beat-to-beat basis. As they directly measure the pressure of arterial blood flow, they are considered to be the gold standard for accuracy in blood pressure measurement [3, 5–9, 11, 12].

As pre-hospital critical care services expand, it may become feasible to establish direct intra-arterial blood pressure monitoring before patients arrive at hospital [1]. However, it is not yet known whether NIBP already provide sufficiently accurate measurements in the pre-hospital critical care setting. The aim of this study was to determine whether NIBP measurements reflect the “true” blood pressure as measured by invasive intra-arterial manometry amongst patients requiring critical care in the pre-hospital environment.

## Methods

A retrospective cohort study was undertaken using routinely collected data from a regional air ambulance organisation.

### Setting

Thames Valley Air Ambulance (TVAA) is a charity providing all Helicopter Emergency Medical Services (HEMS) to critically unwell and injured patients across three counties (Buckinghamshire, Oxfordshire, Berkshire) in England with a total catchment of over 2.1 million people. Clinical teams (typically a pre-hospital emergency medicine doctor and a specialist critical care paramedic) may be dispatched by car or helicopter. TVAA use the RDT Tempus Pro Monitor (Philips, Amsterdam, Netherlands) for NIBP and the TruWave DPT PX600F (Edwards Lifesciences, Irvine, CA, USA) for IBP.

### Data source

The electronic patient record used by TVAA is HEMSbase (Medic One Systems, London, UK), which is an end-to-end clinical tracking database that records vital signs in real-time. Data were extracted directly from the TVAA HEMSbase record.

### Eligibility criteria

All adult patients (aged *≥* 18 years) were included if they were attended by TVAA between 1st May 2020 and 30th April 2023 and had at least one pair of concurrent blood measurements (one NIBP and one IBP) recorded in HEMSbase. Patients were excluded if they did not have paired blood pressure measurements, i.e. NIBP and IBP with the same timestamp. Observations were also marked for exclusion if there was evidence of artefact or the values were implausible, e.g. systolic IBP or NIBP *≤* 0mmHg or > 300mmHg. These observations were then checked against clinical data in HEMSbase and a decision made about their plausibility by two clinical investigators working independently with disagreements adjudicated by a third investigator. All observations were excluded for any patient in whom a single erroneous measurement was identified. This process was completed before any other analyses began.

### Variables and outcomes

The index test was NIPB measurement and the reference standard was IBP measurement using invasive arterial manometry. The primary outcome was “pairwise agreement”, i.e. the proportion of cases in which the index test showed acceptable agreement with the reference standard. An acceptable difference was pre-specified to be < 20mmHg for SBP and DBP, and < 10mmHg for MAP. These are the thresholds used to define clinically significant orthostatic hypotension [13] and senior pre-hospital clinicians within the author group considered this difference to be one that may affect their clinical decisions about a patient. Mean arterial pressure (MAP) was calculated using its standard definition: MAP = (SBP + 2(DBP))/3. Haemodynamic shock was defined as IBP SBP < 90mmHg and severe hypertension as > 160mmHg.

### Statistical analysis

Differences between the index test (NIBP) and reference standard (IBP) were calculated by using the maximum of (NIBP-IBP) and (IBP-NIBP) for each pair of observations.

The method recommended by Bland and Altman [14] was used to construct plots to visualise differences between pairs of data on the y axis versus the arithmetic mean scaled logarithmically on the x-axis [15]. Pairwise differences were tested for normality using the Shapiro-Wilk method [16]. As these were not normally distributed, regression-based estimates of bias and 95% limits of agreement were calculated [17]. In this way, limits of agreement could be included to account for varying standard deviations and graphically show the interval within which 95% of differences between data pairs would be expected to fall.

Limits of agreement determined using the Bland-Altman method are a product of the data itself and do not say anything about what measurement variation is acceptable, which should be determined *a priori* on clinical grounds [18]. As above, an acceptable difference was defined as < 20mmHg for SBP and DBP and < 10mmHg for MAP.

Categorical variables were compared with pairwise agreement using Chi-square (*X*^2^) tests and non-normally distributed continuous variables using Kruskall-Wallis one-way analysis of variance. Multivariable logistic regression models were fitted to examine associations between pairwise agreement (yes/no) as the dependent variable and patient age (as a continuous variable), reason for critical care (medical/non-medical), transport status (transit/stationary), arterial catheter site (radial/femoral), and haemodynamic shock. Haemodynamic shock was replaced in the base model with “severe hypertension” to evaluate associations with this latter variable. All regression models accounted for clustering of observations within individual patients and used robust standard errors. Complete case analyses were undertaken in the event of missing data. The unit of analysis was that of individual blood pressure measurements and not patients.

Statistical analyses were undertaken using Stata 15.0 (College Station, TX, USA) and the threshold for statistical significance set at two-tailed *p* < 0.05. The Shapiro-Wilk test was performed and Bland-Altman plots created using the swilk [19] and blandaltman [15] modules respectively in Stata. The statistical code used for these analyses is included as Supplementary File 1.

### Data governance

As investigators outside the TVAA clinical team only required access to a fully anonymised dataset, research ethics committee was not required as per NHS Governance Arrangements for Research Ethics Committee (GAfREC) guidance [20]. The project was approved by the TVAA Medical and Operations Directors. Data suppression was used to avoid reporting any cells with < 5 observations.

## Results

**Fig. 1:**
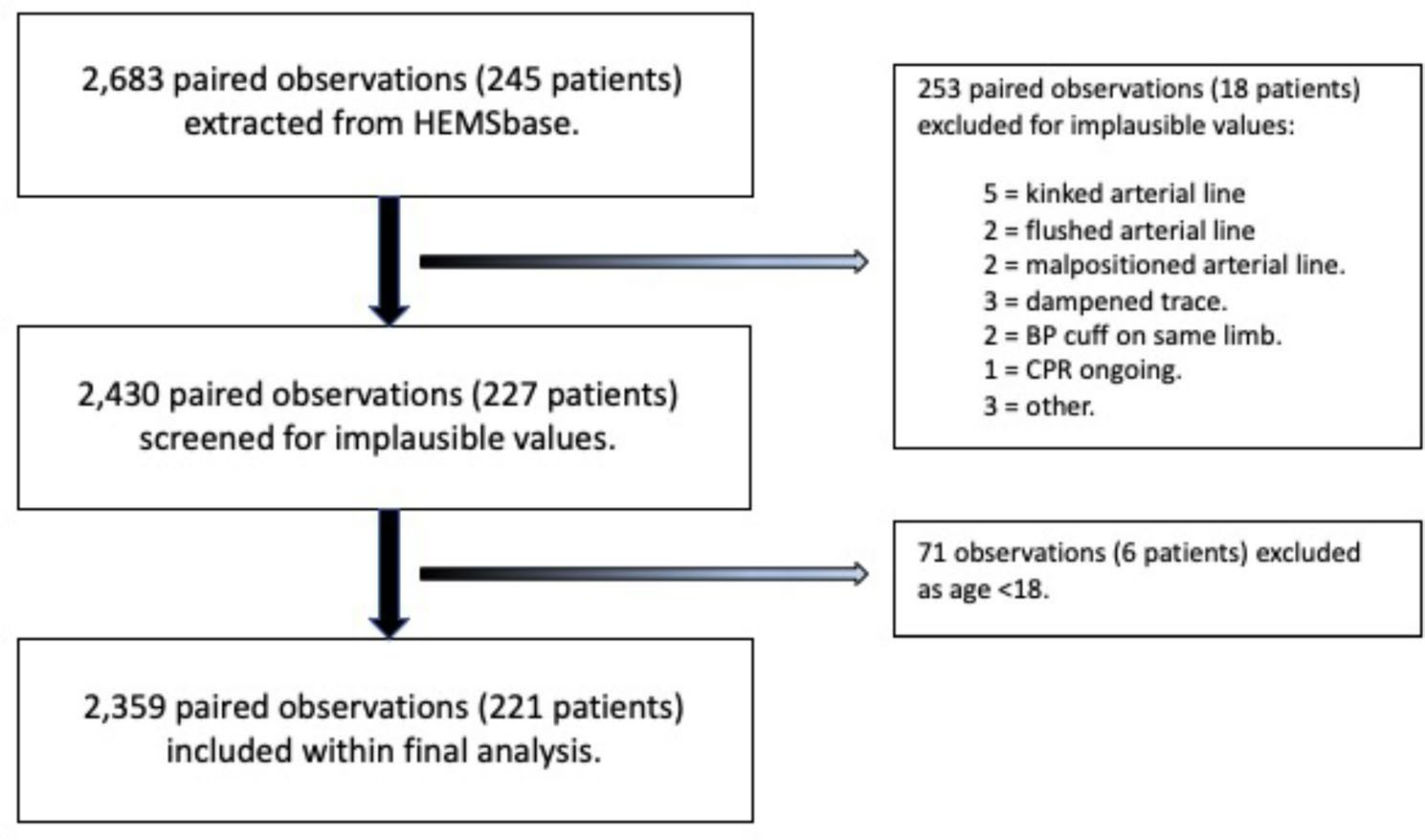
Flow diagram showing reasons for exclusion of observations from the initial dataset

There were 2,683 paired observations in the initial dataset from which 324 were excluded leaving 2,359 paired measurements from 221 individual patients (Fig. 1).

### Cohort description

The median age was 57 (interquartile range 43–72) and the most frequent reasons for requiring pre-hospital critical care were cardiac arrest (*n* = 79, 35.7%) and major trauma (*n* = 83, 37.5%). Arterial catheters were most commonly positioned in a radial artery (*n* = 155, 70.1%) with all others in a femoral artery (*n* = 34, 15.4%), although the location was not recorded in 32 cases (14.5%). Most measurements were undertaken during transport (*n* = 1,668, 70.7%) with the others either before or after the patient had been conveyed to hospital (*n* = 691, 29.3%).

### Index test performance

Table 1 shows the mean blood pressure measurements recorded by both non-invasive and invasive methods as well as the mean difference between the paired observations.

**Table 1:** Mean blood pressure measurements recorded by the index test and reference standard

	Index test (NIBP)	Reference standard (IBP)	Proportion of measurements within acceptability interval
SBP	125.8 (28.3)	130.0 (37.5)	63.8%
DBP	71.7 (21.1)	72.2 (21.4)	75.8%
MAP	96.4 (22.7)	92.9 (26.0)	54.8%

### Bland-Altman analyses

**Fig. 2:**
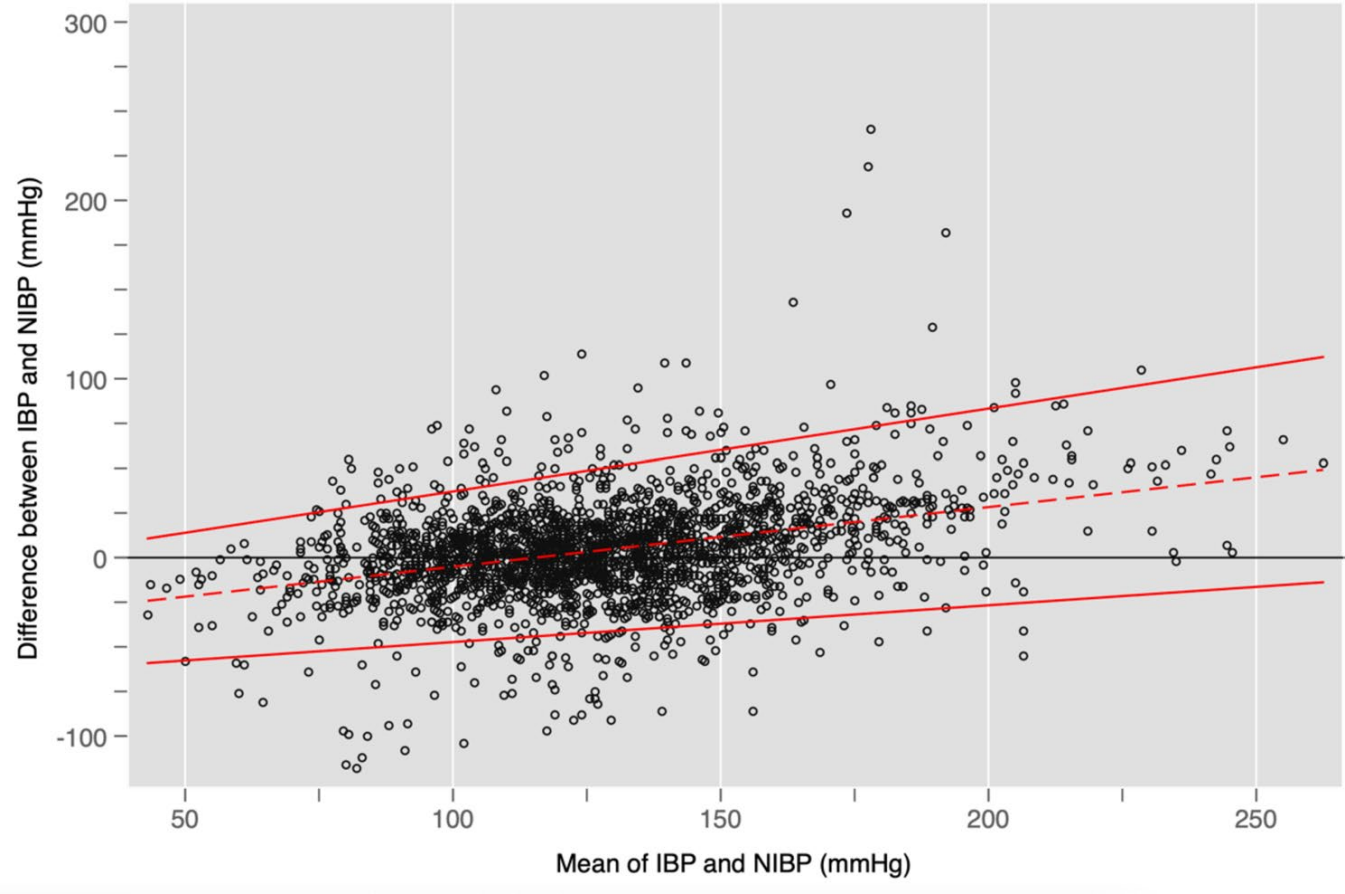
Bland-Altman plot showing differences for each pair of SBP measurements together with bias (dashed red line) and limits of agreement (solid red line). The horizontal black line would reflect perfect agreement between IBP and NIBP at all blood pressures. In this case, the dashed red line slopes upwards, which suggests that the overall bias is towards NIBP *over*estimating SBP during hypotension and *under*estimating SBP during hypertension

**Fig. 3:**
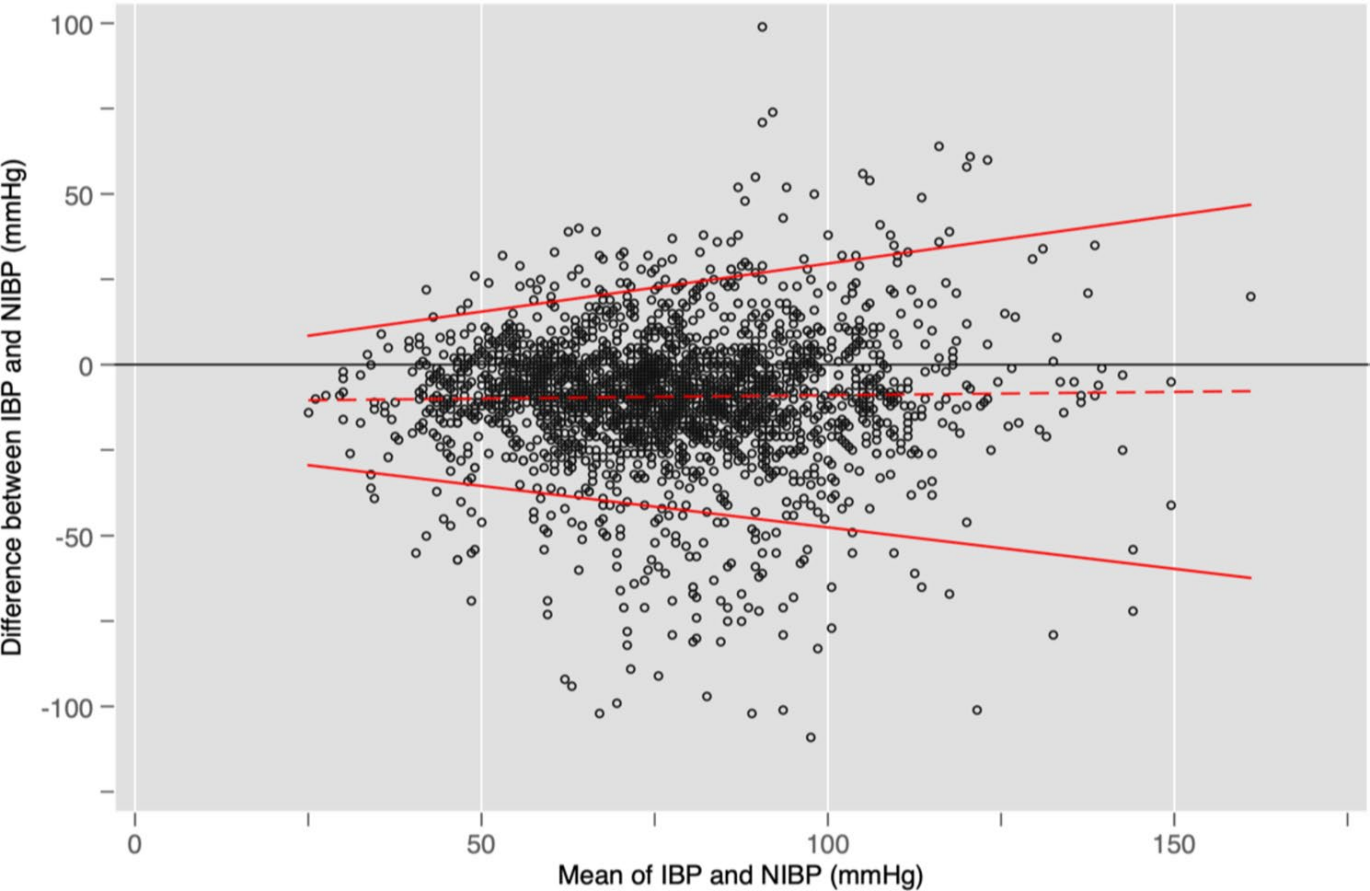
Bland-Altman plot showing differences for each pair of DBP measurements together with bias (dashed red line) and limits of agreement (solid red line). The horizontal black line would reflect perfect agreement between IBP and NIBP at all blood pressures. In this case, the dashed red line runs parallel but below the horizontal black line, which suggests that DBP was consistently overestimated across the whole range of blood pressures

**Fig. 4:**
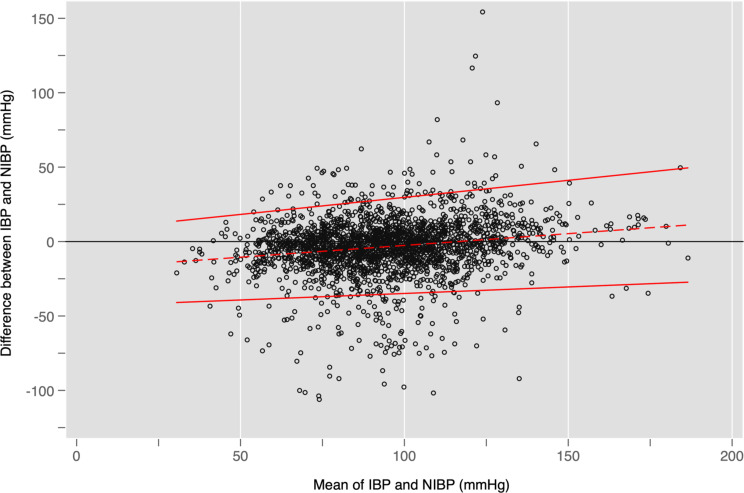
Bland-Altman plot showing differences for each pair of MAP measurements together with bias (dashed red line) and limits of agreement (solid red line). The horizontal black line would reflect perfect agreement between IBP and NIBP at all blood pressures. As in Fig. 2, the dashed red line slopes upwards, which suggests that the overall bias is towards NIBP *over*estimating MAP during hypotension and *under*estimating MAP during hypertension

Figures 2 and 3, and 4 show Bland-Altman plots for SBP, DBP, and MAP respectively. Overall, Figs. 2 and 4 suggest that SBP and MAP are overestimated by NIBP at low values and underestimated at high values. Figure 3 shows that NIBP consistently overestimated DBP across the range of DBP measurements. All three plots showed a broad spread of differences with unacceptably wide 95% limits of agreement that exceeded the pre-specified thresholds of acceptability even at their narrowest points.

### Associations with pairwise agreement

In univariate analysis, age was associated a lower proportion of cases in which the NIBP showed acceptable agreement with IBP for SBP (*p* = 0.004), DBP (*p* < 0.001), and MAP (*p* = 0.008). However, within a multivariable logistic regression model, age was only associated with reduced agreement for DBP (adjusted odds ratio [aOR] 0.99, 95% CI 0.98 to 1.00) but not SBP or MAP (Table 2).

**Table 2:** Adjusted odds of paired blood pressure readings (NIBP vs. IBP techniques) showing acceptable agreement

	Variable	Odds ratio	95% CI	*P*-value
SBP	Age	1.00	0.99 to 1.01	0.432
	Haemodynamic shock	0.52	0.35 to 0.77	0.001
	Severe hypertension	0.17	0.11 to 0.27	< 0.001
	Medical	0.78	0.52 to 1.17	0.237
	Transport	0.89	0.64 to 1.22	0.468
	Femoral line	0.92	0.62 to 1.37	0.685
DBP	Age	0.99	0.98 to 1.00	0.005
	Haemodynamic shock	0.65	0.42 to 0.99	0.047
	Severe hypertension	0.83	0.58 to 1.18	0.301
	Medical	1.28	0.90 to 1.82	0.168
	Transport	0.77	0.57 to 1.03	0.080
	Femoral line	0.66	0.42 to 1.03	0.065
MAP	Age	1.00	0.99 to 1.00	0.330
	Haemodynamic shock	0.53	0.36 to 0.78	0.001
	Severe hypertension	0.82	0.60 to 1.12	0.218
	Medical	1.08	0.78 to 1.48	0.651
	Transport	0.88	0.69 to 1.13	0.324
	Femoral line	0.65	0.46 to 0.91	0.012

Haemodynamic shock was associated with a lower agreement for SBP (50.6% vs. 65.4%, Χ^2^ *p* < 0.001), DBP (67.1% vs. 76.8%, Χ^2^ *p* = 0.001), and MAP (39.8% vs. 56.6%, Χ^2^ *p* < 0.001). Shock was similarly associated with reduced agreement for SBP (aOR 0.52, 95% CI 0.35 to 0.77), DBP (aOR 0.65, 95% CI 0.42 to 0.99) and MAP (aOR 0.53, 95% CI 0.36 to 0.78). By contrast, hypertension was associated with reduced agreement for SBP (31.4% vs. 70.7%, *p* < 0.001) but not DBP (73.9% vs. 76.2%, *p* = 0.321) or MAP (50.6% vs. 55.7%, *p* = 0.059). This pattern persisted within a multivariable regression model: SBP aOR 0.17 (95% CI 0.11 to 0.27), DBP aOR 0.83 (95% CI 0.58 to 1.18), MAP aOR 0.82 (95% CI 0.60 to 1.12).

A medical cause was associated with a lower agreement for SBP (61.7% vs. 67.5%, Χ^2^ *p* = 0.005) but not DBP (76.3% vs. 74.8%, Χ^2^ *p* = 0.428) or MAP (54.3% vs. 55.6%, Χ^2^ *p* = 0.531). Within a multivariable logistic regression model, a medical cause was associated with increased agreement for DBP (aOR 1.49, 95% CI 1.02 to 2.18) but not SBP (aOR 0.73, 95 CI 0.52 to 1.03) or MAP (aOR 1.14, 95% CI 0.81 to 1.59).

Being transported was not associated with reduced agreement for SBP (63.3 vs. 64.8%, *p* = 0.483), DBP (74.6% vs. 78.4%, *p* = 0.050), or MAP (54.1% vs. 56.3%, *p* = 0.338). There was also no association with transport within a multivariable regression model (Table 2).

A femoral arterial line was associated with lower agreement for SBP (59.8% vs. 64.6%, Χ^2^ *p* < 0.001), DBP (68.1% vs. 76.9%, Χ^2^ *p* < 0.001), and MAP (43.9% vs. 56.2%, Χ^2^ *p* < 0.001). However, within a multivariable logistic regression model, a femoral line was only associated with reduced pairwise agreement for MAP (aOR 0.65, 95% CI 0.46 to 0.91) but not SBP (aOR 0.92, 95 CI 0.62 to 1.37) or DBP (aOR 0.66, 95% CI 0.42 to 1.03).

## Discussion

This study found high discordance between NIBP and IBP measurements within a cohort of critically ill and injured patients attended by a pre-hospital critical care service. This is consistent with studies from the anaesthesia [6], intensive care [3, 12], and aeronautical retrieval [7, 8, 11] settings that have reported inaccuracies in NIBP measurements.

This finding is important as pre-hospital critical care clinicians may be initiating treatments including vasopressors or antihypertensives based on these measurements [1]. Their patients may also require tight blood pressure control, e.g. in the case of stroke, severe head injuries, spinal cord injury, post-arrest, and hypertensive emergencies [4]. Delays or failure to recognise changes in blood pressure may have important consequences in this setting [1, 11].

It is a particular concern that NIBP was least reliable amongst hypotensive patients whose blood pressure management is arguably most important [1]. Importantly, this finding was based on a high number of observations (*n* = 261) but from only a small number of individual patients (*n* = 5). However, it is consistent with previous studies that found NIBP to perform least effectively at extremes of blood pressure [3, 5, 6]. Similarly, our data suggest that NIBP underestimated blood pressure in cases of severe hypertension. Anecdotally, TVAA clinicians reported that NIBP devices were more likely to cycle repeatedly but ultimately fail to produce a reading at extremes of blood pressure. Although our study only included recorded measurements and so could not test this observation empirically, this is a further reason to be concerned about relying on NIBP in haemodynamically unstable patients. Future studies should record and compare the ability of NIBP and IBP techniques to produce measurements at the extremes of blood pressure.

Previous studies have found that MAP is more robust as a measurement than SBP and DBP when measured by non-invasive methods [11]. Most oscillometric NIBP devices obstruct arterial flow by inflating a cuff before a pressure sensor is used to detect oscillations in flow as the cuff is deflated [2]. The MAP is measured directly based on the maximum oscillations detected but both SBP and DBP are derived from this figure using an algorithm that varies between manufacturers [1]. As these proprietary algorithms were not derived from patients with critical illness, they may be poorly calibrated for use in the pre-hospital setting. However, our study did not find that MAP was more consistently accurate than SBP or DBP.

This study did not find any evidence that NIBP accuracy was associated with the patient being transported, which is particularly important for pre-hospital services given that oscillometric NIBP measurements may be affected by vibration [7, 8]. Importantly, our study did not distinguish between patients transported by land ambulance or helicopter and it is possible that these conditions vary by mode of transport [21]. This finding is however consistent with an earlier aeromedical transfer study [11] and may suggest that such factors are less important than previously suspected.

Arterial catheters positioned in the femoral artery were associated with lower pairwise agreement than those in the radial artery. This is a significant finding given that our study relied on IBP acting as a reference standard and accurate reflection of the “true” blood pressure. Although errors in arterial measurement may arise due to poor calibration, incorrect zeroing of the transducer, and catheter kinking [22], IBP is widely accepted as the gold standard technique for directly measuring blood pressure [3, 5–9, 11, 12]. However, previous studies in which measurements have been taken from both radial and femoral arteries concurrently have shown that these can vary, particularly in patients receiving inotropes [23, 24]. Femoral artery catheters are positioned more centrally and so may provide a more accurate measure of visceral perfusion [21, 23, 24]. As most of the arterial catheters in this study (70.1%) were positioned in the radial artery, this is a further means by which our data may have overestimated the accuracy of NIBP measurements.

### Limitations

This study has a number of limitations. First, implausible values were excluded before the analyses were undertaken and it is likely that these included the lowest quality measurements using both techniques. This was justified because – in practice – a pre-hospital critical care clinician would not act on measurements that are obviously incorrect. Including such erroneous measurements would likely have had the effect of further reducing pairwise agreement. Second, we pragmatically defined a difference of < 20mmHg (for SBP/DBP) or < 10mmHg (for MAP) as falling within an acceptable range based on what the senior investigators judged would affect treatment within the pre-hospital setting. This choice was more generous that other studies, which used differences of 5 [12, 25] or 10mmHg [4, 11], and so may have overestimated the accuracy of NIBP measurements. The study also presented Bland-Altman plots so that readers can visualise for themselves the variability between NIBP and IBP measurements. Third, the study assumed that all devices were correctly used as measurement accuracy may be affected by practices such as using the wrong sized cuff or applying a cuff over clothing [26]. However, this reflects the use of devices in the pre-hospital setting and may be an important source of real-world variability between NIBP and IBP measurements. Fourth, there is a risk that the measurements reported in this study from a single air ambulance organisation reflect the performance of a narrow range of blood pressure devices. Further studies would be helpful to validate these findings across a wider range of devices within the pre-hospital environment. Finally, this was an observational study and – although we adjusted for known potential confounders – we cannot know the magnitude and direction of biases introduced by residual confounding.

## Conclusion

Non-invasive blood pressure measurements are often inaccurate within the pre-hospital critical care setting, particularly in patients with haemodynamic instability which are the group in which the accuracy of vital signs is most important. In particular, it overestimated SBP and MAP at low values and underestimated them at high values. The limitations of this study would have collectively been expected to *over*estimate the accuracy of NIBP, which makes the low concordance between NIBP and IBP measurements all the more concerning. Pre-hospital clinicians should be aware that NIBP may be misleading and factor this uncertainty into their decision making. Further work may help determine whether direct arterial pressure monitoring should have a wider role in the pre-hospital critical care environment.

### Electronic supplementary material

Below is the link to the electronic supplementary material.

Supplementary Material 1

## Data Availability

Requests to access the anonymised dataset would need to be considered by Thames Valley Air Ambulance as data controller. The statistical code has been made available as a supplementary file.

## Declarations

### Author information

YP is a Specialty Registrar in Emergency Medicine; JR is a Consultant in Emergency and Pre-Hospital Emergency Medicine; KP is a Critical Care Paramedic; DM is the Kadoorie Associate Professor of Emergency Medicine; AL is a Consultant in Anaesthetics and Critical Care Transfer Medicine.
